# Phylogenetic analysis of pectin-related gene families in *Physcomitrella patens* and nine other plant species yields evolutionary insights into cell walls

**DOI:** 10.1186/1471-2229-14-79

**Published:** 2014-03-26

**Authors:** Thomas W McCarthy, Joshua P Der, Loren A Honaas, Claude W dePamphilis, Charles T Anderson

**Affiliations:** 1 Department of Biology, The Pennsylvania State University, University Park, PA 16802, USA

**Keywords:** Plant cell wall, Pectin, *Physcomitrella patens*, *Arabidopsis thaliana*, Phylogeny, Evolution

## Abstract

**Background:**

Pectins are acidic sugar-containing polysaccharides that are universally conserved components of the primary cell walls of plants and modulate both tip and diffuse cell growth. However, many of their specific functions and the evolution of the genes responsible for producing and modifying them are incompletely understood. The moss *Physcomitrella patens* is emerging as a powerful model system for the study of plant cell walls. To identify deeply conserved pectin-related genes in *Physcomitrella*, we generated phylogenetic trees for 16 pectin-related gene families using sequences from ten plant genomes and analyzed the evolutionary relationships within these families.

**Results:**

Contrary to our initial hypothesis that a single ancestral gene was present for each pectin-related gene family in the common ancestor of land plants, five of the 16 gene families, including homogalacturonan galacturonosyltransferases, polygalacturonases, pectin methylesterases, homogalacturonan methyltransferases, and pectate lyase-like proteins, show evidence of multiple members in the early land plant that gave rise to the mosses and vascular plants. Seven of the gene families, the UDP-rhamnose synthases, UDP-glucuronic acid epimerases, homogalacturonan galacturonosyltransferase-like proteins, β-1,4-galactan β-1,4-galactosyltransferases, rhamnogalacturonan II xylosyltransferases, and pectin acetylesterases appear to have had a single member in the common ancestor of land plants. We detected no *Physcomitrella* members in the xylogalacturonan xylosyltransferase, rhamnogalacturonan I arabinosyltransferase, pectin methylesterase inhibitor, or polygalacturonase inhibitor protein families.

**Conclusions:**

Several gene families related to the production and modification of pectins in plants appear to have multiple members that are conserved as far back as the common ancestor of mosses and vascular plants. The presence of multiple members of these families even before the divergence of other important cell wall-related genes, such as cellulose synthases, suggests a more complex role than previously suspected for pectins in the evolution of land plants. The presence of relatively small pectin-related gene families in *Physcomitrella* as compared to *Arabidopsis* makes it an attractive target for analysis of the functions of pectins in cell walls. In contrast, the absence of genes in *Physcomitrella* for some families suggests that certain pectin modifications, such as homogalacturonan xylosylation, arose later during land plant evolution.

## Background

Pectins make up approximately one third of the dry mass of primary cell walls in eudicots, affecting both water dynamics and the mechanical behavior of the wall [1]. Pectins consist of four domains: homogalacturonan (HG), xylogalacturonan (XGA), rhamnogalacturonan I (RG-I), and rhamnogalacturonan II (RG-II) [2]. Homogalacturonan makes up the majority of the pectic component of the cell wall and also serves as the backbone of XGA and RG-II. Xylogalacturonan is made up of HG with attached xylose side-groups, whereas RG-II has four complex and distinct side-chains [3]. Rhamnogalacturonan I has side-chains containing galactose and arabinose, but its backbone consists of alternating rhamnose and galacturonic acid. These complex polysaccharides are almost universally conserved in land plants and are also present in some algae [4], although structural diversity in pectins is present between some species. For instance, there is evidence for RG-II in all land plant species analyzed to date [3,5] but its side chains are not perfectly conserved [6], and the side chains of RG-I vary among species [1]. Additionally, XGA has not been detected in *Physcomitrella patens*[7].

Pectins are important determinants of wall remodeling during cellular growth [8]. Pairs of HG molecules can be bound together by Ca^2+^ bridges, stiffening the wall [9], and RG-II side-chains dimerize via borate diol ester bonds [10]. A decreased ability to form RG-II dimers leads to dwarfism [11]. Modifications to pectin can enhance or prevent these interactions and thus affect the properties of the wall as a whole: for example, alterations in wall stiffness mediated by pectin methylation have been implicated in organ primordium initiation and cell elongation [8,12]. Pectins also appear to be essential for normal cell-cell adhesion, since some pectin methylation-defective mutants lack tissue cohesion [13,14].

The complex structures of pectins require a large suite of biosynthetic genes, many of which are inferred only by the biochemical reactions required to synthesize the many linkages in pectins [15,16]. Nevertheless, many pectin-related genes have been identified, and modification of their expression can have serious effects on the development and growth of mutant plants [17-20]. Pectins play an especially important role in the tip growth of pollen tubes, with methylation status regulating the yielding properties of the tip and side walls [21,22], but this system does not allow for easy genetic manipulation. *Physcomitrella patens*, the model moss [23], represents an attractive experimental system for the genetic and molecular analysis of pectins in the walls of tip-growing cells. Its primary growth form is a mass of protonemal filaments that extend exclusively via tip growth and might therefore rely heavily on pectins for normal development [24,25]. Genes in the *Physcomitrella* genome [26] can be modified directly using high-efficiency homologous recombination [27], which, combined with the dominant haploid generation of this moss, makes it ideal for genetic modification and analysis. As a moss, *Physcomitrella* is also likely to resemble an early stage in the transition of plants from aquatic to terrestrial life, giving us a clearer view of the cell wall architectures and physiology that made this transition possible.

**Figure 1:**
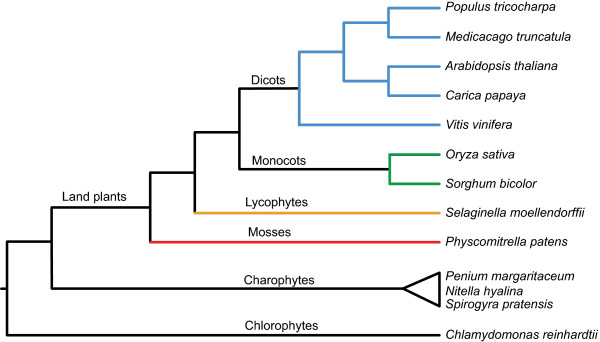
**Summary of land plant phylogeny.** The evolutionary relationships of the ten PlantTribes species used in this study (land plants and *Chlamydomonas*) and the charophycean algae used as additional outgroups. Note that only one moss and one lycophyte genome has been sequenced to represent early-diverging lineages of land plants, compared with many genomes representing angiosperms.

As diverse plant genomes are sequenced, there are new opportunities to study gene families in an evolutionary context. The PlantTribes 2.0 database [28] is an objective gene family classification that can be used to investigate gene family composition and phylogeny on a global scale. By using the complete inferred protein sequences from ten diverse plant genomes (seven angiosperms plus the lycophyte *Selaginella moellendorffii*, the moss *Physcomitrella*, and the chlorophyte *Chlamydomonas reinhardtii;* see Figure 1), orthologous gene clusters (orthogroups) were identified that represent deeply conserved, but often narrowly defined gene families. Orthogroups were constructed using OrthoMCL [29], resulting in gene clusters that typically align well across their length and have a conserved domain structure [30]. Leveraging the PlantTribes 2.0 classification is a conservative approach to identify gene family members from sequenced genomes, avoiding false positive hits that may be identified using less structured search algorithms (e.g. BLAST). To assess the complexity of the pectin biosynthetic and modification machinery in *Physcomitrella* and to investigate the evolutionary history of pectin-related gene families in land plants, we performed an orthogroup-based phylogenetic study of 16 gene families associated with pectin production and modification and mapped the relationships of these genes among terrestrial plant species with sequenced genomes. These analyses reveal that the *Physcomitrella* genome contains at least one member in most of the families analyzed and that the total number of pectin-related gene family members in *Physcomitrella* is much lower than that in *Arabidopsis*. Analysis of these families not only identified members in *Physcomitrella*, it also reveals that several pectin-related gene families likely had multiple members in the land-plant common ancestor.

## Results

### Identification of pectin-related genes using PlantTribes 2.0

We used a set of genes in *Arabidopsis* belonging to 16 pectin-related gene families identified in the literature (Additional file 1) to select orthogroups in the PlantTribes 2.0 database for in-depth phylogenetic analysis (Additional file 2) [28]. The number of genes from each species in each family is displayed in Additional file 3. We found at least one *Physcomitrella* gene in 12 of the 16 families examined (Table 1). Notably, no *Physcomitrella* members of the xylogalacturonan xylosyltransferase (Additional file 4), rhamnogalacturonan-I arabinosyltransferases (Additional file 5), pectin methylesterase inhibitor (Additional file 6), or polygalacturonase inhibitor protein (Additional file 7) families were detected. There were fewer *Physcomitrella* members in most of the pectin-related gene families than in *Arabidopsis*, with the exception of the UDP-rhamnose synthase (four *Arabidopsis*, six *Physcomitrella*), β-1,4-galactan β-1,4-galactosyltransferase (three *Arabidopsis*, four *Physcomitrella*), and UDP-glucuronic acid (UDP-GlcA) epimerase (five *Arabidopsis*, nine *Physcomitrella*) families.

**Table 1:** **Representatives of pectin-related gene families in *Arabidopsis *** **and *Physcomitrella***

**Pectin-related gene family**	***Arabidopsis *** **genes**	***Physcomitrella *** **genes**	**Putative minimum # of family members in common ancestor**
UDP-Rhamnose synthases	4	**6**	1
UDP-Glucuronic acid epimerases	5	**9**	1
Galacturonosyltransferases (GAUTs)	**15**	8	3
GAUT-like proteins (GATLs)	**10**	3	1
β-1,4-Galactan β-1,4-Galactosyltransferase	3	**4**	1
Rhamnogalacturonan II xylosyltransferases	**4**	1	1
Rhamnogalacturonan I arabinosyltransferases	**2**	0	ND
Xylogalacturonan xylosyltransferases	**2**	0	ND
Homogalacturonan methyl-transferases	**6**	3	2
Pectin methylesterases	**66**	14	5
Pectin methylesterase inhibitors (PMEIs)	**2**	0	ND
Polygalacturonases	**67**	10	5
Polygalacturonase Inhibitor Proteins (PGIPs)	**2**	0	ND
Pectate lyase-like proteins	**26**	7	2
Pectin acetylesterases	**11**	1	1
Pectin acetyltransferases	**4**	3	1
Totals	229	69	24

Sixteen gene families were analyzed. For each gene family, the number under the species with the larger number of genes is highlighted in bold. In most cases there were more *Arabidopsis* members than *Physcomitrella* members. ND (not determined); phylogenetic ambiguity prevents an accurate estimation of ancestral gene number at this time.

### Phylogenetic analysis of pectin-related gene families

**Figure 2:**
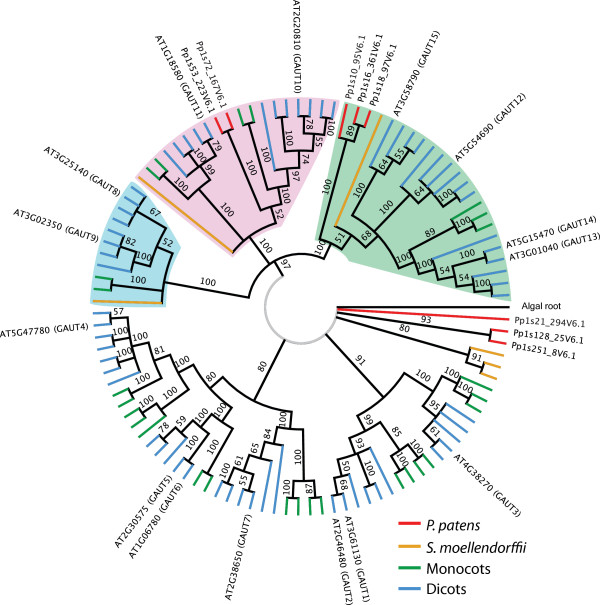
**GAUT family tree.** Three well-supported clades that suggest ancestral GAUTs are highlighted (blue, pink, and green clouds), and an unresolved polytomy near the root of the tree is indicated in light grey. The green and pink clades, as well as the polytomy, contain monocot, eudicot, *Selaginella*, and *Physcomitrella* members, whereas the blue clade does not have any *Physcomitrella* members. The algal root gene from *Spirogyra pratensis* falls within the polytomy.

**Figure 3:**
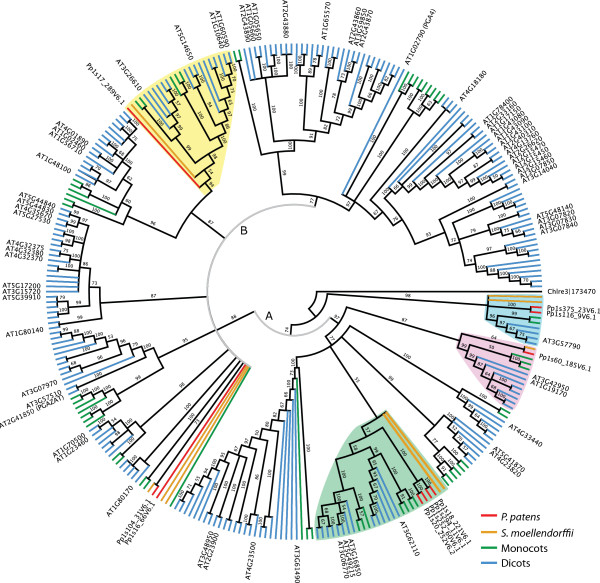
**Polygalacturonase family tree.** Four monophyletic clades (blue, pink, green, and yellow clouds) contain monocot, eudicot, *Selaginella*, and *Physcomitrella* genes. The tree contains two large polytomies, indicated in light grey and labeled “A” and “B”. Polytomy B contains unresolved *Physcomitrella* and *Selaginella* members. The algal root gene is from *C. reinhardtii*, a chlorophytic alga.

**Figure 4:**
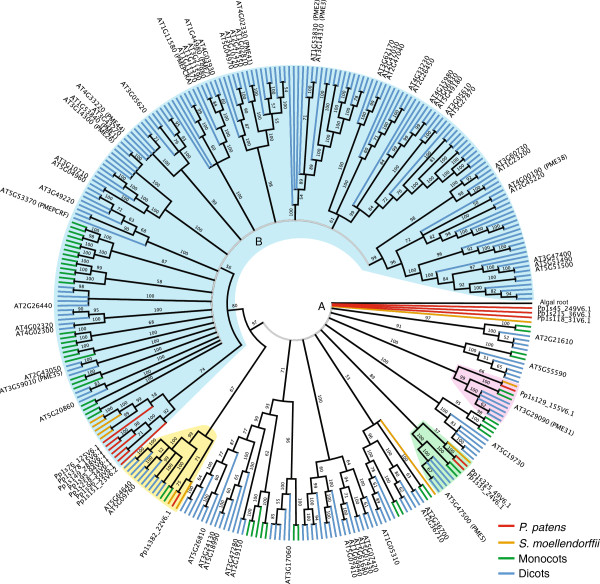
**Pectin-methylesterase family tree.** Two large polytomies, labeled “A” and “B” and shown in light grey, indicate poor resolution of some of this family’s lineages. Four monophyletic clades contain members from the monocots, eudicots, *Selaginella*, and *Physcomitrella*. One of these clades (blue cloud) consists of polytomy B and a smaller clade of *Physcomitrella* and *Selaginella* genes. Additional moss and tracheophyte genes remain poorly resolved in polytomy A. The algal root (from *P. margaritaceum*) is within one of the polytomies.

Our identification of pectin-related genes in ten diverse plant species (Figure 1) provided an opportunity to examine their phylogenetic patterns [31]. To analyze the evolutionary relationships between gene family members, we aligned the sequences from the PlantTribes 2.0 search results for each family using the MUSCLE algorithm [32] followed by manual curation, and constructed maximum likelihood trees from these alignments using RAxML [33]. Where possible, we also included a homologous gene from a green alga to root the trees. We tested the hypothesis that each pectin-related gene family would trace back to a single ancestral gene in the common ancestor of land plants, with any *Physcomitrella* genes forming a clade sister to all other land plants. Surprisingly, this was the case for only seven of the 16 families examined (Table 1). Five of the trees have multiple well-supported land plant-wide clades (Figures 2, 3, 4, Additional file 8 and Additional file 9). Each clade is evidence for a separate ancestral gene in the early land plant ancestor of the terrestrial species examined. These trees and their implications are explored below.

### The *GAUT* superfamily contains at least five ancestral land plant genes

The GAUT superfamily consists of the GAUT and the distantly-related GAUT-like (GATL) families [34,35]. Some galacturonosyltransferases (GAUTs) are responsible for constructing HG and use UDP-galacturonic acid (UDP-GalA) as a substrate [34]. In *Arabidopsis*, mutations in GAUTs cause phenotypes ranging from changes in sugar composition of the wall to severe dwarfism to apparent lethality [34,36-38]. In our analysis, the GAUT family tree contains three large well-resolved clades, as well as an unresolved polytomy (Figure 2). Genes from *Physcomitrella* and tracheophytes are present in two of these clades and within the polytomy from which the root algal gene is not resolved. The third of these clades includes genes from *Selaginella*, monocots, and eudicots but no *Physcomitrella* genes. This tree suggests a minimum of four ancestral GAUTs in the earliest land plant.

**Figure 5:**
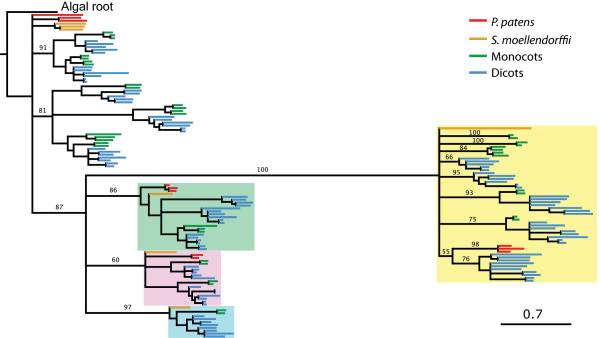
**GAUT superfamily tree.** In this tree, phylogenetic distance is indicated by branch length. The GATL gene family (yellow cloud) is well-supported as being derived from within the GAUTs; due to a polytomy in the GATL family, clade relationships within this family are not well resolved. The distance of the GATLs from the GAUTs suggests an ancient divergence, but the position of the algal root supports the hypothesis that the GATLs descended from the GAUTs rather than diverging from a common ancestor. Scale bar, 0.7 substitutions/site.

The roles of the GATL proteins are not all clearly established: some of them have been implicated in pectin production, while at least one seems to be involved in xylan synthesis [38,39]. When we generated an alignment and phylogenetic tree of the entire superfamily (Figure 5), the *GATL* family (yellow cloud) appeared as a well-resolved but distant clade derived from within the *GAUT* family that also contains representatives from all of the land plant species queried.

### Polygalacturonase and pectin methylesterase families are large and deeply conserved

Whereas GAUTs build the HG backbone of pectins, polygalacturonases (PGs) hydrolyze it, weakening the pectin matrix and potentially loosening the wall [40]. In eudicots, PGs are important in cell expansion and also in abscission and fruit softening [41]. The PG family is very large in *Arabidopsis*, with over 65 known members. Our phylogenetic analysis for these genes resulted in two large unresolved polytomies, each containing several monophyletic groups, four of which contain representatives from mosses, lycophytes, monocots, and eudicots (Figure 3). Although the placement of several of the *Physcomitrella* genes is unresolved, the gene tree suggests a minimum of five genes in the common ancestor.

Like the PGs, the pectin methylesterase (PME) family is very large in *Arabidopsis*[42]. Galacturonic acid residues in the HG backbones of pectins often have attached methyl ester groups at the C6 position that can prevent pectin-modifying enzymes as well as interactions with other HG chains. Thus, the amount and pattern of methylation can affect wall dynamics in several ways. PMEs remove methyl groups from pectin, rendering it more prone to degradation by hydrolytic enzymes as well as to calcium cross-linking, potentially either weakening or stiffening the wall. This is complicated by the tendency of different PMEs to remove methyl groups in random or block-wise patterns: lone de-methylated GalAs make the polymer prone to enzyme degradation, whereas consecutive exposed carboxylate groups favor calcium-bridging [43]. Like the PGs, the PME gene tree we generated has two large polytomies and two smaller resolved clades (Figure 4). Unlike the PG tree, the algal root is a member of one of the polytomies. Within this polytomy are two well-supported land plant-wide monophyletic clades. Resolved from this polytomy is a third land plant-wide clade. Several *Physcomitrella* and *Selaginella* genes are in a clade that is sister to the second polytomy, which consists entirely of angiosperm genes. This tree suggests that a minimum of five PMEs existed in the common ancestor of the species examined.

### Many pectin-related gene families appear to have had only one or two members in the common ancestor of land plants

Like the polygalacturonases, pectate lyase-like proteins cleave the HG backbone of pectins (Additional file 8) [44]. Homogalacturonan methyltransferases are responsible for methylating newly synthesized HG (Additional file 9) [13]. Both of these family trees indicate the existence of multiple members in the common ancestor by having multiple supported clades with members from every division of the plant lineage. The final seven of the family trees have *Physcomitrella* genes grouped sister to the other land plants, indicating a single ancestral gene prior to the divergence of *Physcomitrella* and the tracheophytes: the UDP-GlcA epimerases, the UDP-rhamnose synthases, the pectin acetylesterases, the pectin acetyltransferases, the RG-II xylosyltransferases, the β-1,4-galactan β-1,4-galactosyltransferases, and the GATLs (Additional files 10, 11, 12, 13, 14, 15 and 16). These families are listed as having one supported common ancestral gene in Table 1. The UDP-GlcA epimerase, UDP-rhamnose synthase, β-1,4-galactan β-1,4-galactosyltransferase, and GATL families all likely expanded in *Physcomitrella* after its divergence from the tracheophytes.

## Discussion

### Search and tree-building criteria for pectin-related genes

We adopted a relatively stringent set of criteria to identify putative orthologs of *Arabidopsis* pectin-related genes in *Physcomitrella* and other plant species, and used these genes to build phylogenetic trees of pectin-related gene families. Rather than simply using database searches and overall sequence similarity to identify homologous genes, we leveraged the network of global gene relationships in the PlantTribes 2.0 database to identify clusters of orthologous genes (orthogroups) from the other species for analysis. Using BLAST to identify putative gene orthologs is a common practice, but increases the number of false positive sequences obtained because hits may only share high similarity in a small portion of the gene (i.e. a conserved domain), but may not be closely related and align poorly across the full length of the sequence. In contrast to BLAST-based methods, the use of PlantTribes 2.0 orthogroups increases the probability of identifying genes within the same evolutionary lineage, thus reflecting the history of these gene families more accurately. In some cases our search method detected fewer *Physcomitrella* members than other analyses of these families [40,45,46]. In all of these cases the researchers used shared protein domains or sequence homology to identify their genes of interest. The search method we used was intended to identify high-confidence candidate genes for further experimental analysis that are more likely to share conserved functions within other model systems. We therefore employed a higher-stringency approach at the cost of missing more distantly related homologs.

Although our trees largely agree with previously published phylogenies for some pectin-related gene families [35,36,40,45-49], the larger number of species we used improved our ability to resolve gene family topologies and to detect basal branchpoints that have been obscured in analyses using genome data from fewer species [36,40,46-49]. An exception to this is the work of Wang *et al*., which identified PMEs and PMEIs in the same land plant species we examined, as well as *Amborella trichopoda*[45]. Wang *et al*. searched for conserved PME and PMEI protein domains and identified 35 putative *Physcomitrella* PMEs as compared with our ten. They also produced a large PMEI tree that included a putative *Physcomitrella* member. In contrast to our approach, their domain-based approach likely resulted in the detection of distantly related genes not included in our results.

### Several pectin-related gene families likely had multiple members in the common ancestor of mosses and tracheophytes

The topologies of the trees we generated provide clues to the evolutionary relationships between known pectin-related genes and their orthologs in other species. This allows us to hypothesize about the state of the gene families in the last common ancestor of *Physcomitrella* and vascular plants. In seven of the families we analyzed, the paralogs in *Physcomitrella* are sister to all other genes in vascular plants. On the other hand, several of the families (GAUTs, HG methyltransferases, PMEs, PGs, pectate lyase-like proteins) each appear to have had multiple members in the common ancestor of land plants. Our analyses suggest that the suite of genes for the production, modification, and degradation of pectins had already diversified prior to the radiation of land plants. This contrasts with the cellulose synthase gene family (CESA), which likely contained a single gene in the ancestor of land plants and subsequently diversified after the divergence of mosses and vascular plants [50]. Multiple members of a gene family often have different expression patterns, allowing for tissue-specific regulation of the associated activity; for example, PpCESA5 is required only for gametophore development, implying that other PpCESAs produce cellulose in protonemal tissue [51]. Intriguingly, others have hypothesized that pectin synthesis and modification might originally have been central in wall production and modulation, with the importance of cellulose arising later [52]. There is also evidence for further diversification of these families before the flowering plant divergence in the form of angiosperm-wide clades in the GAUTs, PMEs, PGs, pectate lyase-like proteins, UDP-glucuronic acid epimerases, UDP-rhamnose synthases, and pectin acetylesterases.

### Some pectin-related gene families were not detected in *Physcomitrella*

Since orthogroups in the PlantTribes 2.0 database generally represent narrowly defined gene lineages that typically align well across the whole length of the gene, we are confident that distantly related genes have been excluded from our analyses. However, it is possible that we failed to detect highly divergent members of some of these gene families. Nevertheless, most of the searches yielded at least one *Physcomitrella* gene per family. This was not true of the XGA xylosyltransferases, the RG I arabinosyltransferases, the PGIPs, and the PMEIs. It is not surprising that XGA xylosyltransferases were not detected in *Physcomitrella* given that a previous study using comprehensive microarray polymer profiling (COMPP) did not detect XGA in *Physcomitrella* cell walls [7]. On the other hand, α(1–5)-arabinans characteristic of RG I were detected in the pectic fraction of *Physcomitrella* walls, which combined with the failure to detect *Physcomitrella* orthologs of *AtARAD* genes in this study and others [49] raises the possibility of the existence of other arabinan-arabinosyltransferases that are only distantly related to the currently known genes.

Although there are not any studies indicating that PGIPs are absent in *Physcomitrella*, we also did not detect any PGIP genes in *Selaginella*, suggesting that this gene family may have evolved after the divergence of lycophytes and euphyllophytes. PGIPs are thought to play a role in pathogen defense by preventing foreign PGs from degrading the plant cell wall [53], and it is interesting that none were detected in either our representative moss or lycophyte, given that *Physcomitrella* and other mosses are susceptible to fungal pathogens [54]. The PMEI tree we generated only contains genes from *Arabidopsis* and *Medicago truncatula*, and might not adequately represent the diversity in this gene family. This might be due to insufficient numbers of query genes to allow for the detection of all the family members, or because coding sequence information for some of the species might have been incomplete. Importantly, the *Arabidopsis* query genes were both contained within one orthogroup. Genome data for additional plant species and/or future improvements in genome annotations could potentially overcome this limitation.

### *Arabidopsis* has an abundance of pectin-related genes, whereas grasses appear to have fewer pectin-related genes in some families

In nine of the 16 families analyzed, *Arabidopsis* had more members than any of the other species (Additional file 3). This might be the result of the more extensive annotation of the *Arabidopsis* genome as compared to other species in the database, or the unique genome duplication histories of the species analyzed [30]. We see a general trend of more pectin-related genes in the eudicots than in the monocots and more in the monocots than in the more basal species such as *Physcomitrella* and *Selaginella*. This may reflect the lower levels of pectin in the walls of grasses compared to other flowering plants [55], as well as the relatively high abundance of other acidic polymers such as glucuronoarabinoxylans in grasses [56]. Further phylogenetic analyses of non-commelinid monocots, which have Type I cell walls [57], might be informative in determining the relationship between the elaboration of pectin-related gene families and the abundance of pectins in the cell wall.

## Conclusions

Pectins play a key role in the cell walls of plants. We analyzed 16 gene families involved in the production, modification, and degradation of pectins in nine land plant species. Our analysis indicates that although many of these families appear to trace back to a single gene in the last common ancestor to the mosses and the vascular plants, several of the major families involved in pectin regulation likely contained multiple genes. We did not detect *Physcomitrella* or *Selaginella* genes in four of the studied families, providing some evidence that they might have evolved after the divergence of seed plants from the lycophytes. This study has allowed us to identify *Physcomitrella* orthologs related to known pectin-related genes in *Arabidopsis* for in-depth experimental analysis. Our results also shed light on the evolutionary history of pectin biosynthesis and modification, suggesting that pectins may have played an important role in the transition from an aquatic to a terrestrial environment.

## Methods

### Identification of pectin-related gene families

We compiled a list of *Arabidopsis* genes with known and predicted pectin-related functions using TAIR and Uniprot annotations, as well as relevant literature (Additional file 1) [1,34,42,53,58-64]. In total, we used 108 genes from *Arabidopsis* to identify putative pectin-related gene families in the PlantTribes 2.0 database [65]. PlantTribes 2.0 is an objective gene family classification of protein coding genes from ten sequenced green plant genomes that have been clustered into orthogroups (putatively monophyletic gene lineages) using OrthoMCL [28]. Orthogroups containing pectin-related genes from *Arabidopsis* were extracted for phylogenetic analysis. This approach enabled us to include additional homologous genes from *Arabidopsis* not annotated with pectin-related gene functions. In some cases, the pectin-related query genes from *Arabidopsis* did not belong to an orthogroup (i.e., they were singletons). The closest *Physcomitrella* gene to each singleton *Arabidopsis* gene was identified via TBLASTX and added to the family alignment. Because PlantTribes 2.0 includes the *Physcomitrella patens* version 1.1 gene annotations from Phytozome [66], we used a nucleotide BLAST+ search of a local database of *Physcomitrella patens* version 1.6 annotated coding sequences to identify the current gene annotations for ease of reference (Additional file 2, which includes all of the genes used in this paper). Although PlantTribes 2.0 does include the chlorophyte alga *Chlamydomonas reinhardtii*, many of the gene families still lacked a non-land plant outgroup. To enhance the possibility of rooting our trees using an outgroup, we also included homologous transcript sequences from three additional green algae (*Nitella hyalina*, *Penium margaritaceum*, and *Spirogyra pratensis*) where possible [67]. We searched each transcriptome separately with coding sequences from *Physcomitrella* using TBLASTX with an E-value cutoff of 10^-10^. Full-length coding sequences were identified for the GAUT, pectin methylesterase, UDP-rhamnose synthase, rhamnogalacturonan I arabinosyltransferase, and rhamnogalacturonan II xylosyltransferase families.

### Phylogenetic analysis

Sequences for each family were aligned by translation in Geneious using MUSCLE (default parameters) [32], manually curated, and saved as relaxed Phylip files (Additional files 17, 18, 19, 20, 21, 22, 23, 24, 25, 26, 27, 28, 29, 30, 31, 32 and 33). In some cases this required removing non-homologous genes and gene fragments from poorly annotated genomes. To generate trees (Additional files 34, 35, 36, 37, 38, 39, 40, 41, 42, 43, 44, 45, 46, 47, 48, 49 and 50), maximum likelihood phylogenetic analysis was performed using RAxML [33] with the following parameters: rapid bootstrap analysis and search for best-scoring maximum likelihood tree in one run, GTRGAMMA model of nucleotide evolution, random seed 12345, 1000 bootstrap replicates. Nodes with less than 50% bootstrap support were collapsed using TreeCollapserCL4 [68] and were visualized using FigTree [69]. Figures were manually edited for readability using Adobe Illustrator.

## Availability of supporting data

The data sets supporting the results of this article are included within the article and its additional files.

## Competing interests

The authors declare that they have no competing interests.

## Authors' contributions

TWM contributed to experimental design, collected query sequences, performed the database searches, identified algal roots, performed the sequence alignments, ran the phylogenetic analyses, prepared the figures, and participated in drafting the manuscript. JPD contributed to experimental design, assisted in sequence alignment, and participated in drafting the manuscript. LAH contributed to experimental design, assisted in sequence alignment, and participated in drafting the manuscript. CWD contributed to experimental design and participated in drafting the manuscript. CTA contributed to experimental design and participated in drafting the manuscript. All authors read and approved the final manuscript.

## Supplementary Material

Additional file 1: Table S1 Query *Arabidopsis* genes. A list of all the *Arabidopsis* genes used as queries to the PlantTribes 2.0 database and the sources for collecting them. Click here for file

Additional file 2: Table S2 Pectin-related genes. This table contains all of the genes examined in this study. Click here for file

Additional file 3: Table S3 Species distribution by family. Plant Tribes 2.0 species list, with the number of pectin-related genes found in each. Click here for file

Additional file 4: Figure S1 Xylogalacturonan xylosyltransferase family tree. *Physcomitrella* and *Selaginella* genes were not detected in this family. Click here for file

Additional file 5: Figure S2 Rhamnogalacturonan I arabinosyltransferase family tree. This tree contains no *Physcomitrella* members and two algal members, one from *Penium margaritaceum* and one from *Nitella hyalina*. Click here for file

Additional file 6: Figure S3 Pectinmethylesterase inhibitor (PMEI) family tree. This tree contains only *Arabidopsis* and *Medicago trunculata* members and likely does not represent the whole family. Click here for file

Additional file 7: Figure S4 Polygalacturonase inhibitor protein family tree. *Physcomitrella* and *Selaginella* genes were not detected in this family. Monocot and eudicot family members are contained in separate clades that are well-resolved from each other. Click here for file

Additional file 8: Figure S5 Pectate lyase-like (PLL) family tree. A small land plant-wide clade is resolved from the rest of the tree (pink cloud), indicating at least two genes in the common ancestor of land plants. Click here for file

Additional file 9: Figure S6 Homogalacturonan methyltransferase family tree. This tree consists of three monophyletic clades, two of which are land plant-wide. An algal root with reasonably homology was not detected for this gene family, preventing the determination of whether two or three ancestral genes were present in the common ancestor of land plants. Click here for file

Additional file 10: Figure S7 UDP-Glucuronic acid epimerase family tree. This family appears to be land plant-wide and is rooted by a gene from *C. reinhardtii*. However, the grouping of all the *Physcomitrella* genes into one monophyletic clade implies that there was only one family member in the common ancestor. Click here for file

Additional file 11: Figure S8 UDP-Rhamnose synthase family tree. Not only is this family land plant-wide, it includes members from the algae *C. reinhardtii*, *Spirogyra pratensis*, and *Penium margaritaceum*, but the grouping of all the *Physcomitrella* genes into one monophyletic clade implies that there was only one family member in the common ancestor. Click here for file

Additional file 12: Figure S9 Pectin acetyltransferase family tree. This family appears to be land plant-wide and is rooted by a gene from *C. reinhardtii*. The grouping of all the *Physcomitrella* genes into one monophyletic clade implies that there was only one family member in the common ancestor. Click here for file

Additional file 13: Figure S10 Pectin acetylesterase family tree. This family contains only one *Physcomitrella* and no *Selaginella* members. Click here for file

Additional file 14: Figure S11 Rhamnogalacturonan II xylosyltransferase family tree. This family appears to be land plant-wide, with one member in the common ancestor of land plants. The algal root gene is from *Nitella hyalina*. Click here for file

Additional file 15: Figure S12 β-1,4-Galactan β-1,4-Galactosyltransferase family tree. This tree has no algal root. The *Physcomitrella* genes are grouped together in a well-supported clade separate from other species. There is no evidence for more than one gene in the common ancestor. Click here for file

Additional file 16: Figure S13 GATL family tree. This tree is poorly resolved, with no root and large polytomies. The *Physcomitrella* genes group together in one well-supported clade. Click here for file

Additional file 17 **galactangalactosyltransferasefamilyalignment.phy.** Phylip gene alignment, .phy. Raw β-1,4-galactan β-1,4-galactosyltransferase alignment. β-1,4-galactan β-1,4-galactosyltransferase family alignment file. Click here for file

Additional file 18 **GATLfamilyalignment.phy.** Phylip gene alignment, .phy. Raw GATL alignment. GATL family alignment file. Click here for file

Additional file 19 **GAUTfamilyalignment.phy.** Phylip gene alignment, .phy. Raw GAUT alignment. GAUT family alignment file. Click here for file

Additional file 20 **GAUTsuperfamilyalignment.phy.**Phylip gene alignment, .phy. Raw GAUT superfamily alignment. GAUT superfamily alignment file. Click here for file

Additional file 21 **homogalacturonanmethyltransferasefamilyalignment.phy.** Phylip gene alignment, .phy. Raw homogalacturonan methyltransferase alignment. Homogalacturonan methyltransferase family alignment file. Click here for file

Additional file 22 **pectatelyaselikefamilyalignment.phy.** Phylip gene alignment, .phy. Raw pectate lyase-like alignment. Pectate lyase-like family alignment file. Click here for file

Additional file 23 **pectinacetylesterasefamilyalignment.phy.** Phylip gene alignment, .phy. Raw pectin acetylesterase alignment. Pectin acetylesterase family alignment file. Click here for file

Additional file 24 **pectinacetyltransferasefamilyalignment.phy.** Phylip gene alignment, .phy. Raw pectin acetyltransferase alignment. Pectin acetyltransferase family alignment file. Click here for file

Additional file 25 **PGIPfamilyalignment.phy.** Phylip gene alignment, .phy. Raw polygalacturonase inhibitor protein alignment. Polygalacturonase inhibitor protein family alignment file. Click here for file

Additional file 26 **PMEfamilyalignment.phy.** Phylip gene alignment, .phy. Raw pectin methylesterase alignment. Pectin methylesterase family alignment file. Click here for file

Additional file 27 **PMEIfamilyalignment.phy.** Phylip gene alignment, .phy. Raw pectin methylesterase inhibitor alignment. Pectin methylesterase inhibitor family alignment file. Click here for file

Additional file 28 **polygalacturonasefamilyalignment.phy.** Phylip gene alignment, .phy. Raw polygalacturonase alignment. Polygalacturonase family alignment file. Click here for file

Additional file 29 **RGIarabinosyltransferasefamilyalignment.phy.** Phylip gene alignment, .phy. Raw rhamnogalacturonan I arabinosyltransferase alignment. Rhamnogalacturonan I arabinosyltransferase family alignment file. Click here for file

Additional file 30 **RGIIxylosyltransferasefamilyalignment.phy.** Phylip gene alignment, .phy. Raw rhamnogalacturonan II xylosyltransferase alignment. Rhamnogalacturonan II xylosyltransferase family alignment file. Click here for file

Additional file 31 **UDPGlcAepimerasefamilyalignment.phy.** Phylip gene alignment, .phy. Raw UDP-glucuronic acid epimerase alignment. UDP-glucuronic acid epimerase family alignment file. Click here for file

Additional file 32 **UDPrhamnosesynthasefamilyalignment.phy.** Phylip gene alignment, .phy. Raw UDP-rhamnose synthase alignment. UDP-Rhamnose synthase family alignment file. Click here for file

Additional file 33 **xylogalacturonanxylosyltransferasefamilyalignment.phy.** Phylip gene alignment, .phy. Raw xylogalacturonan xylosyltransferase alignment. Xylogalacturonan xylosyltransferase family alignment file. Click here for file

Additional file 34 **galactangalactosyltransferase.tree.** Newick tree, .tree. Raw β-1,4-galactan β-1,4-galactosyltransferase tree. β-1,4-galactan β-1,4-galactosyltransferase family tree file with bootstrap values. Click here for file

Additional file 35 **GATL.tree.** Newick tree, .tree. Raw GATL tree. GATL family tree file with bootstrap values. Click here for file

Additional file 36 **GAUT_superfamily.tree.** Newick tree, .tree. Raw GAUT superfamily tree. GAUT superfamily tree file with bootstrap values. Click here for file

Additional file 37 **GAUT.tree.** Newick tree, .tree. Raw GAUT tree. GAUT family tree file with bootstrap values. Click here for file

Additional file 38 **homogalacturonanmethyltransferase.tree.** Newick tree, .tree. Raw homogalacturonan methyltransferase tree. Homogalacturonan methyltransferase family tree file with bootstrap values. Click here for file

Additional file 39 **pectatelyaselike.tree.** Newick tree, .tree. Raw pectate lyase-like tree. Pectate lyase-like family tree file with bootstrap values. Click here for file

Additional file 40 **pectinacetylesterase.tree.** Newick tree, .tree. Raw pectin acetylesterase tree. Pectin acetylesterase family tree file with bootstrap values. Click here for file

Additional file 41 **pectinacetyltransferase.tree.** Newick tree, .tree. Raw pectin acetyltransferase tree. Pectin acetyltransferase family tree file with bootstrap values. Click here for file

Additional file 42 **PGIP.tree.** Newick tree, .tree. Raw polygalacturonase inhibitor protein tree. Polygalacturonase inhibitor protein family tree file with bootstrap values. Click here for file

Additional file 43 **PME.tree.** Newick tree, .tree. Raw pectin methylesterase tree. Pectin methylesterase family tree file with bootstrap values. Click here for file

Additional file 44 **PMEI.tree.** Newick tree, .tree. Raw pectin methylesterase inhibitor tree. Pectin methylesterase inhibitor family tree file with bootstrap values. Click here for file

Additional file 45 **polygalacturonase.tree.** Newick tree, .tree. Raw polygalacturonase tree. Polygalacturonase family tree file with bootstrap values. Click here for file

Additional file 46 **RGIarabinosyltransferase.tree.** Newick tree, .tree. Raw rhamnogalacturonan I arabinosyltransferase tree. Rhamnogalacturonan I arabinosyltransferase family tree file with bootstrap values. Click here for file

Additional file 47 **RGIIxylosyltransferase.tree.** Newick tree, .tree. Raw rhamnogalacturonan II xylosyltransferase tree.Rhamnogalacturonan II xylosyltransferase family tree file with bootstrap values. Click here for file

Additional file 48 **UDPGlcAepimerase.tree.** Newick tree, .tree. Raw UDP-glucuronic acid epimerase tree. UDP-glucuronic acid epimerase family tree file with bootstrap values. Click here for file

Additional file 49 **UDPrhamnosesynthase.tree.** Newick tree, .tree. Raw UDP-rhamnose synthase tree. UDP-Rhamnose synthase family tree file with bootstrap values. Click here for file

Additional file 50 **xylogalacturonanxylosyltransferase.tree.** Newick tree, .tree. Raw xylogalacturonan xylosyltransferase tree. Xylogalacturonan xylosyltransferase family tree file with bootstrap values. Click here for file
