# Supplementary figures and images for: Phylogenetic analysis of pectin-related gene families in Physcomitrella patens and nine other plant species yields evolutionary insights into cell walls
Source: BMC Plant Biol. 2014 Mar 26;14:79. doi: 10.1186/1471-2229-14-79 (PMC4108027; doi:10.1186/1471-2229-14-79)

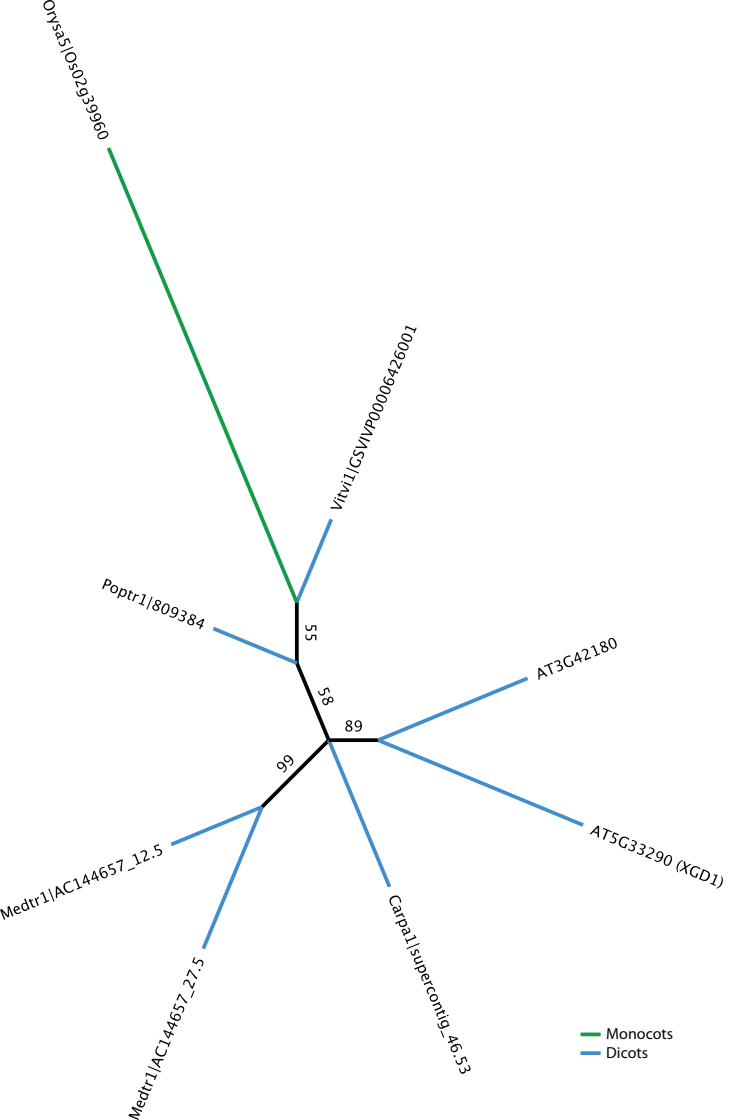

Supplement: Additional file 4: Figure S1 — Xylogalacturonan xylosyltransferase family tree. Physcomitrella and Selaginella genes were not detected in this family. [file 1471-2229-14-79-S4.pdf]

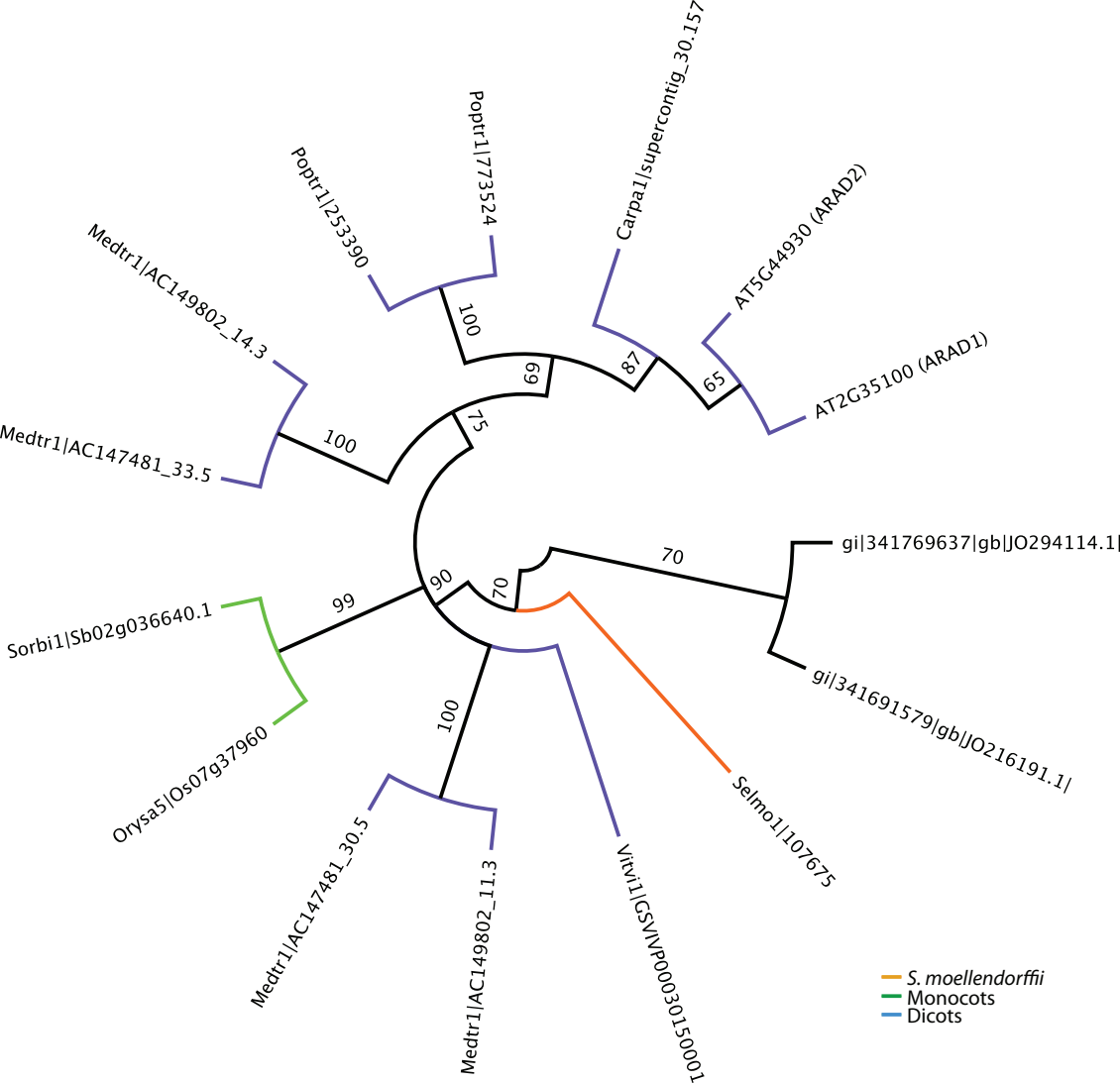

Supplement: Additional file 5: Figure S2 — Rhamnogalacturonan I arabinosyltransferase family tree. This tree contains no Physcomitrella members and two algal members, one from Penium margaritaceum and one from Nitella hyalina. [file 1471-2229-14-79-S5.pdf]

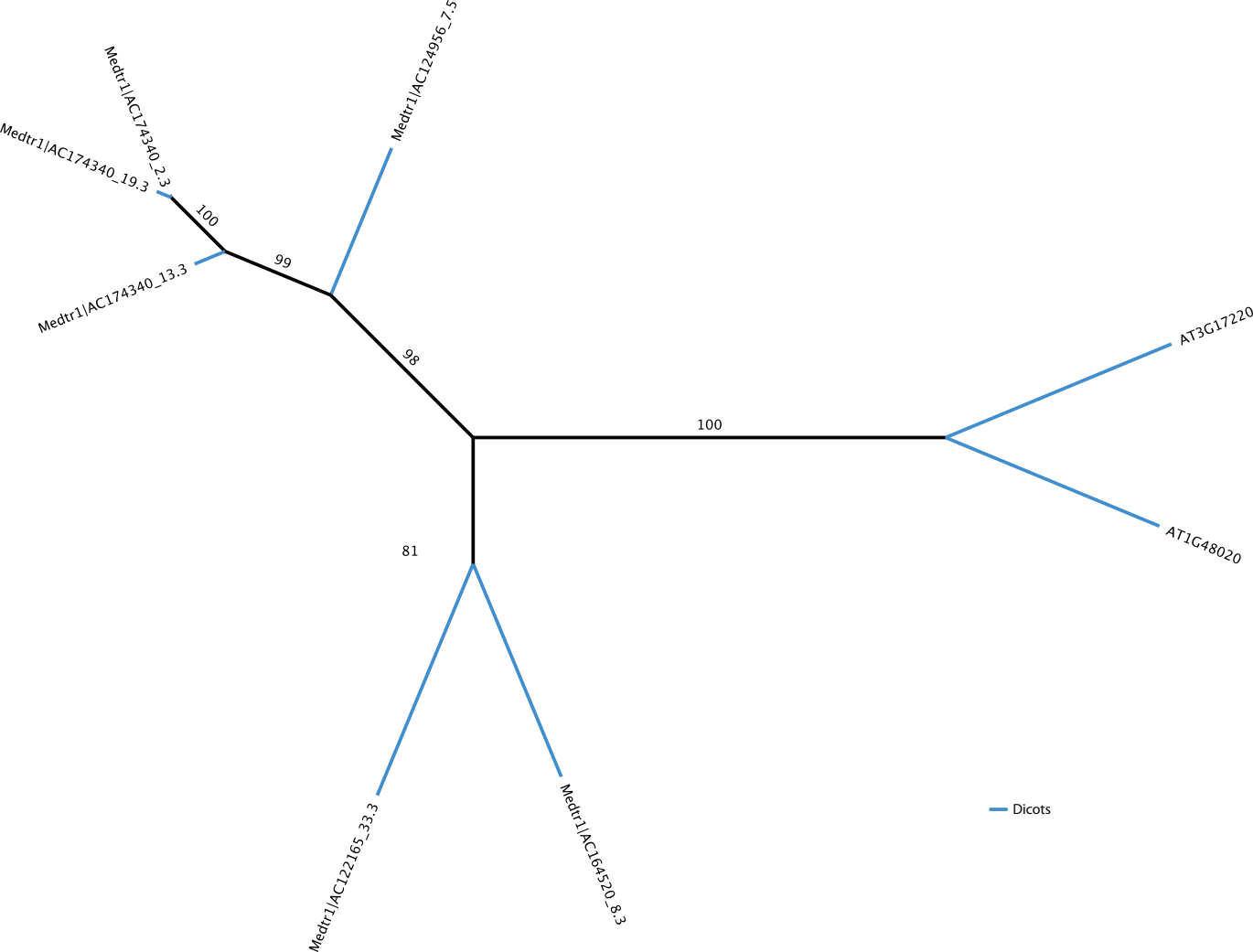

Supplement: Additional file 6: Figure S3 — Pectinmethylesterase inhibitor (PMEI) family tree. This tree contains only Arabidopsis and Medicago trunculata members and likely does not represent the whole family. [file 1471-2229-14-79-S6.pdf]

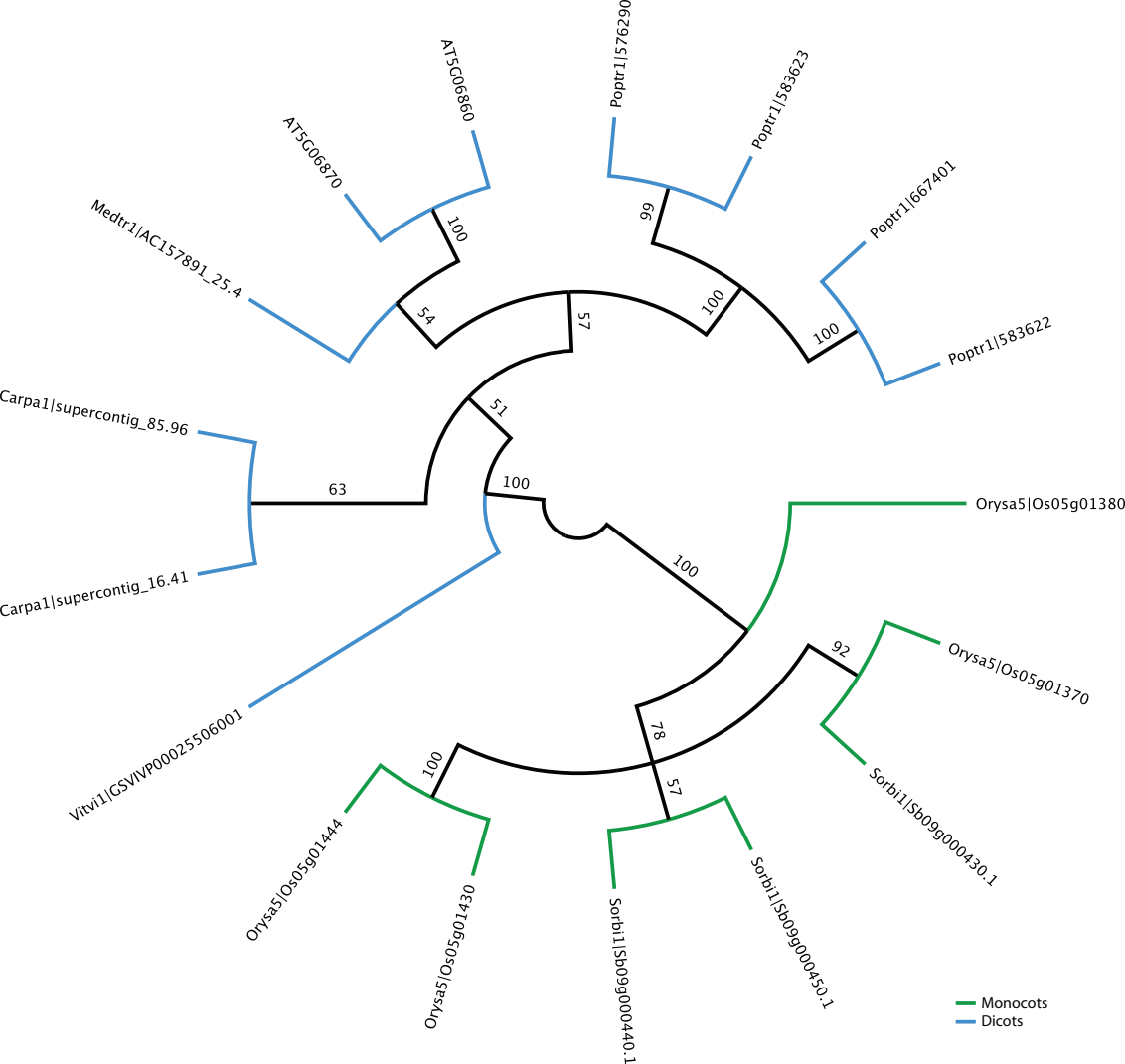

Supplement: Additional file 7: Figure S4 — Polygalacturonase inhibitor protein family tree. Physcomitrella and Selaginella genes were not detected in this family. Monocot and eudicot family members are contained in separate clades that are well-resolved from each other. [file 1471-2229-14-79-S7.pdf]

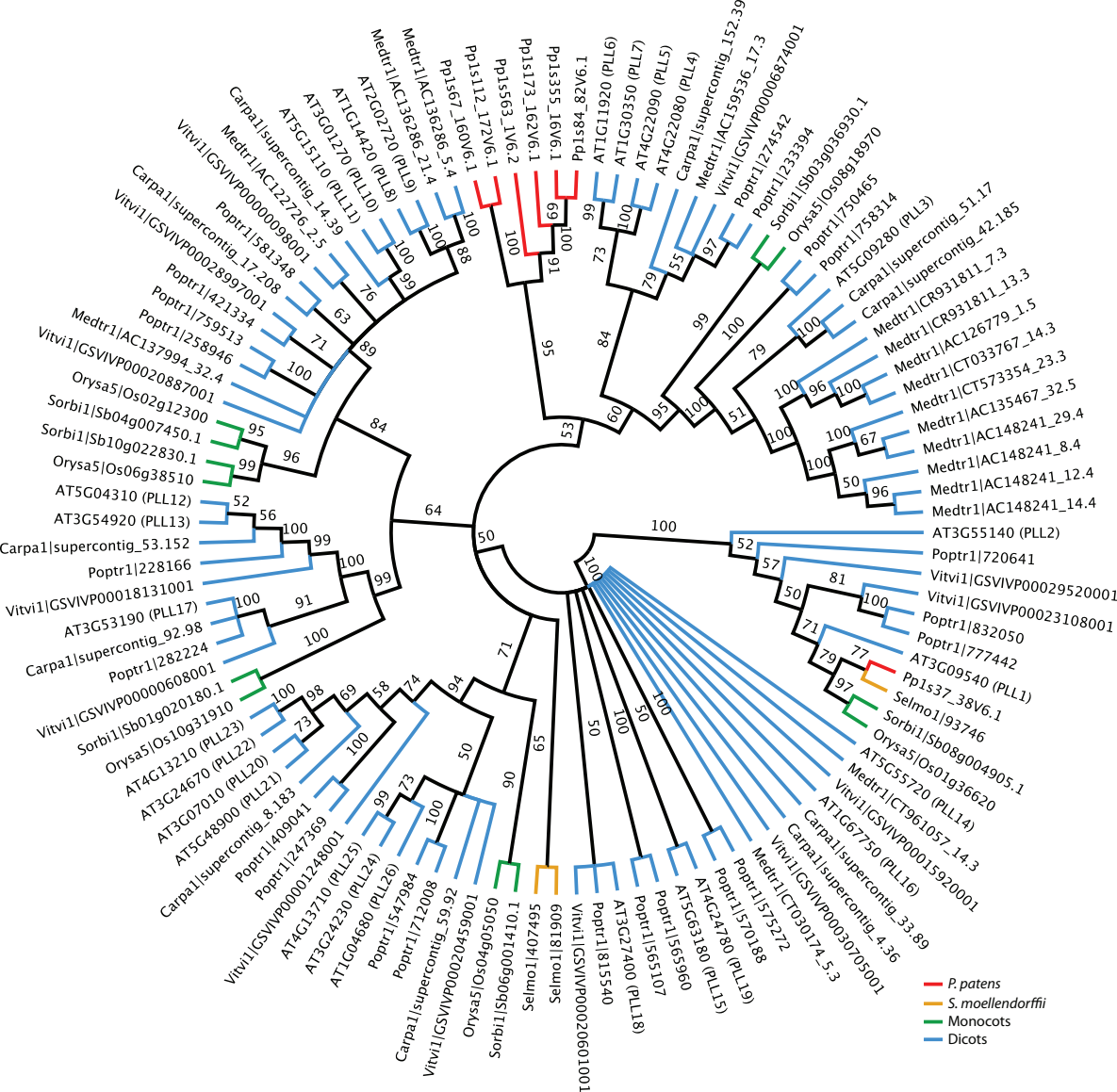

Supplement: Additional file 8: Figure S5 — Pectate lyase-like (PLL) family tree. A small land plant-wide clade is resolved from the rest of the tree (pink cloud), indicating at least two genes in the common ancestor of land plants. [file 1471-2229-14-79-S8.pdf]

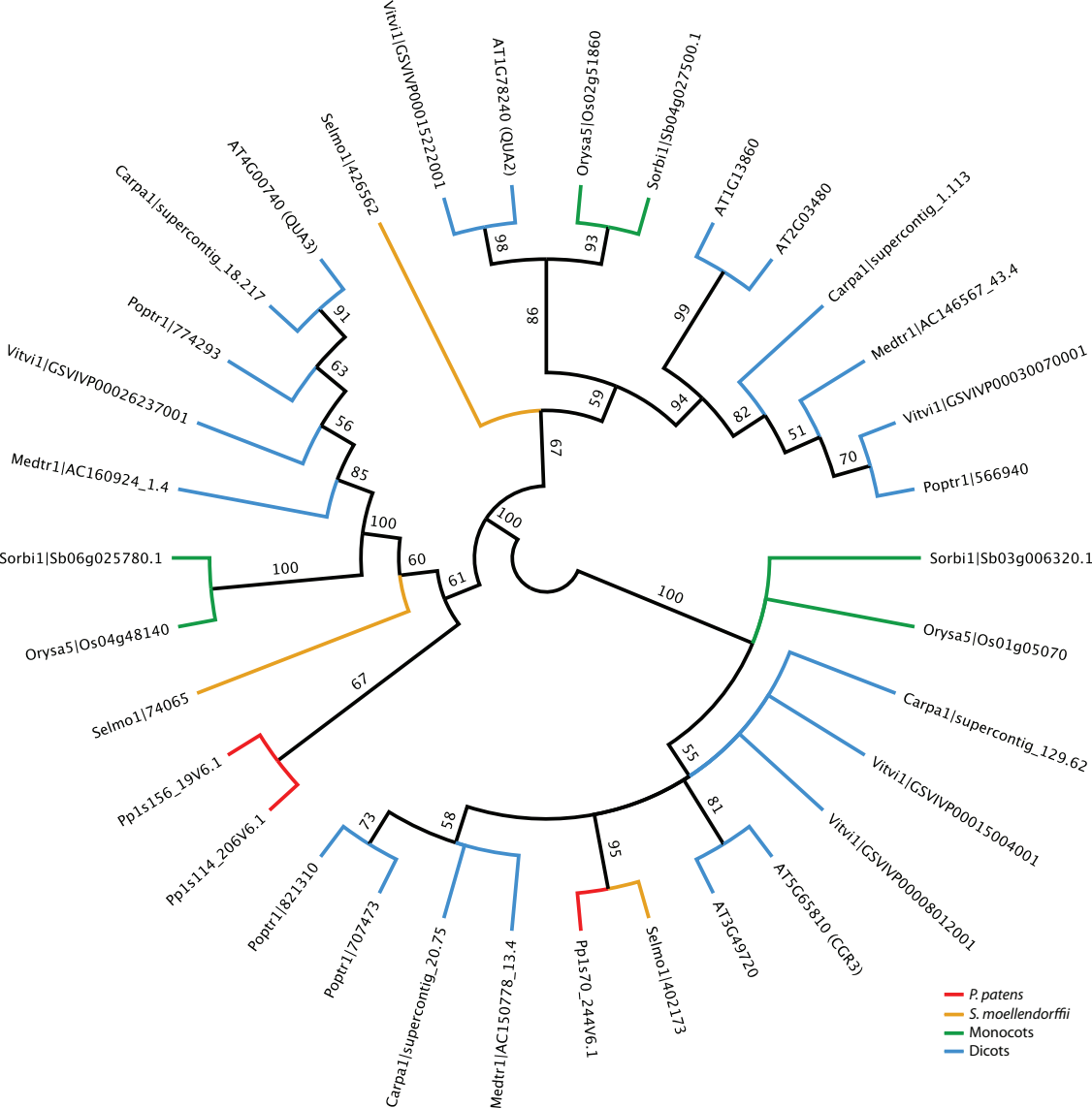

Supplement: Additional file 9: Figure S6 — Homogalacturonan methyltransferase family tree. This tree consists of three monophyletic clades, two of which are land plant-wide. An algal root with reasonably homology was not detected for this gene family, preventing the determination of whether two or three ancestral genes were present in the common ancestor of land plants. [file 1471-2229-14-79-S9.pdf]

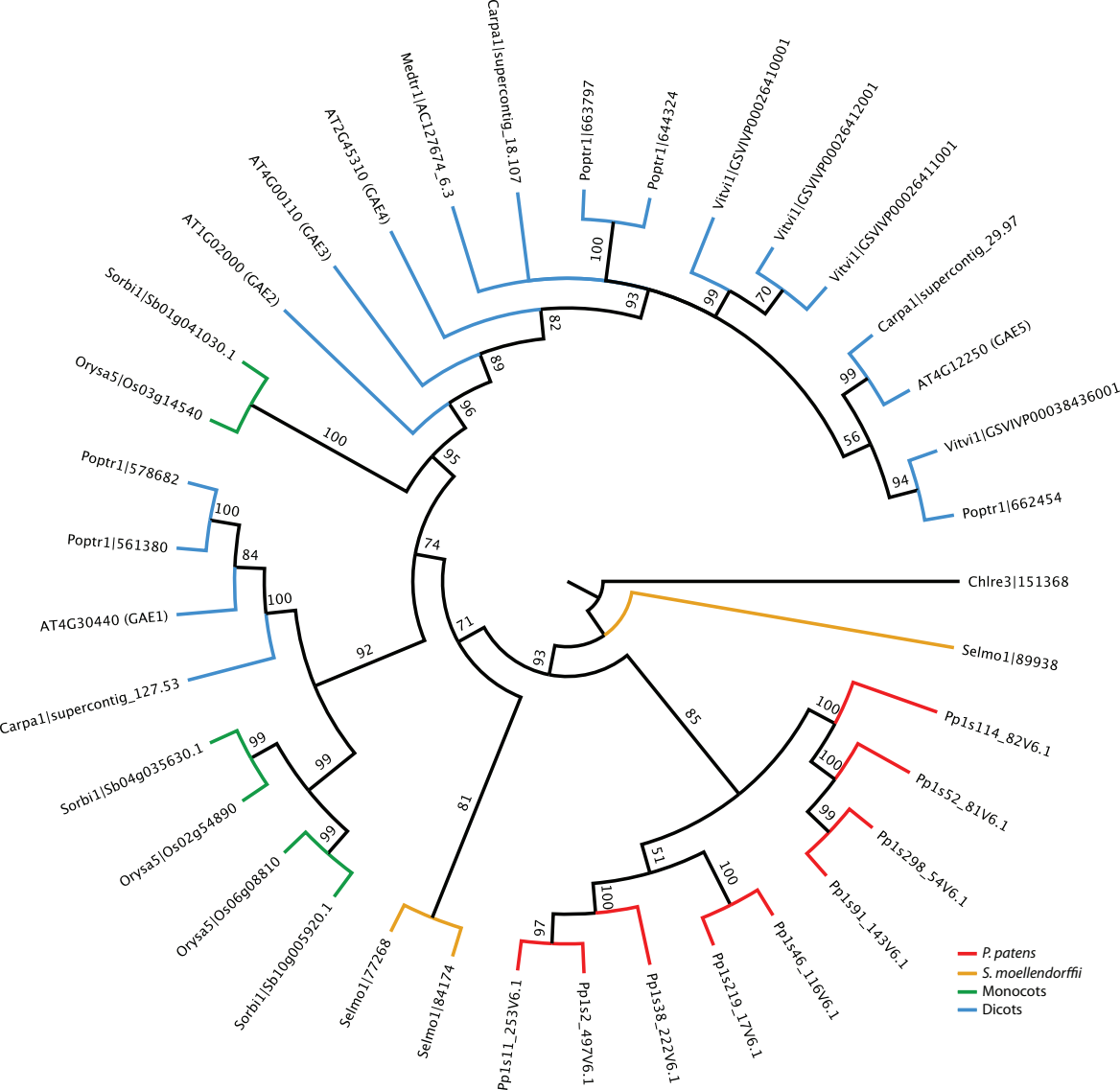

Supplement: Additional file 10: Figure S7 — UDP-Glucuronic acid epimerase family tree. This family appears to be land plant-wide and is rooted by a gene from C. reinhardtii. However, the grouping of all the Physcomitrella genes into one monophyletic clade implies that there was only one family member in the common ancestor. [file 1471-2229-14-79-S10.pdf]

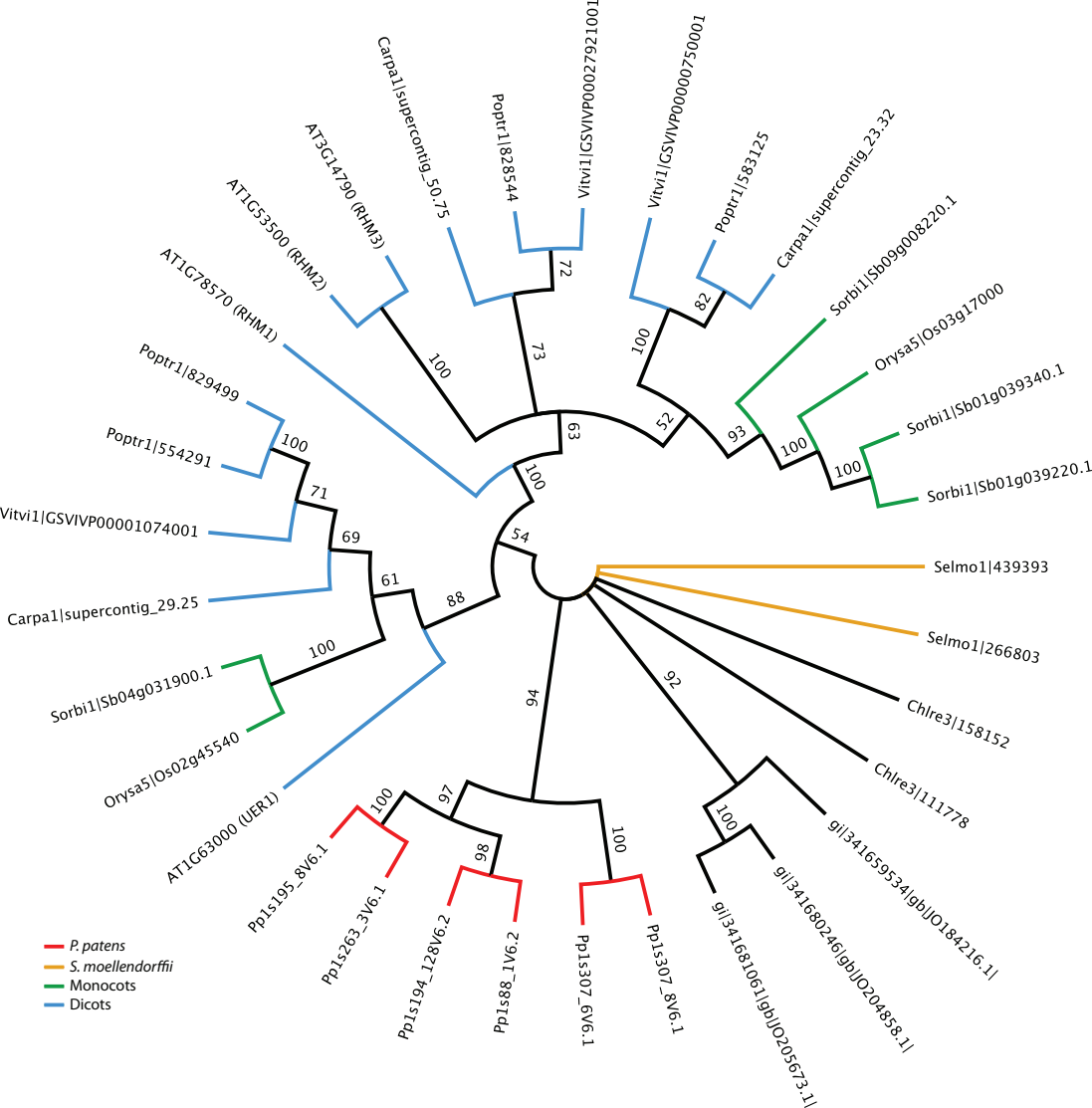

Supplement: Additional file 11: Figure S8 — UDP-Rhamnose synthase family tree. Not only is this family land plant-wide, it includes members from the algae C. reinhardtii, Spirogyra pratensis, and Penium margaritaceum, but the grouping of all the Physcomitrella genes into one monophyletic clade implies that there was only one family member in the common ancestor. [file 1471-2229-14-79-S11.pdf]

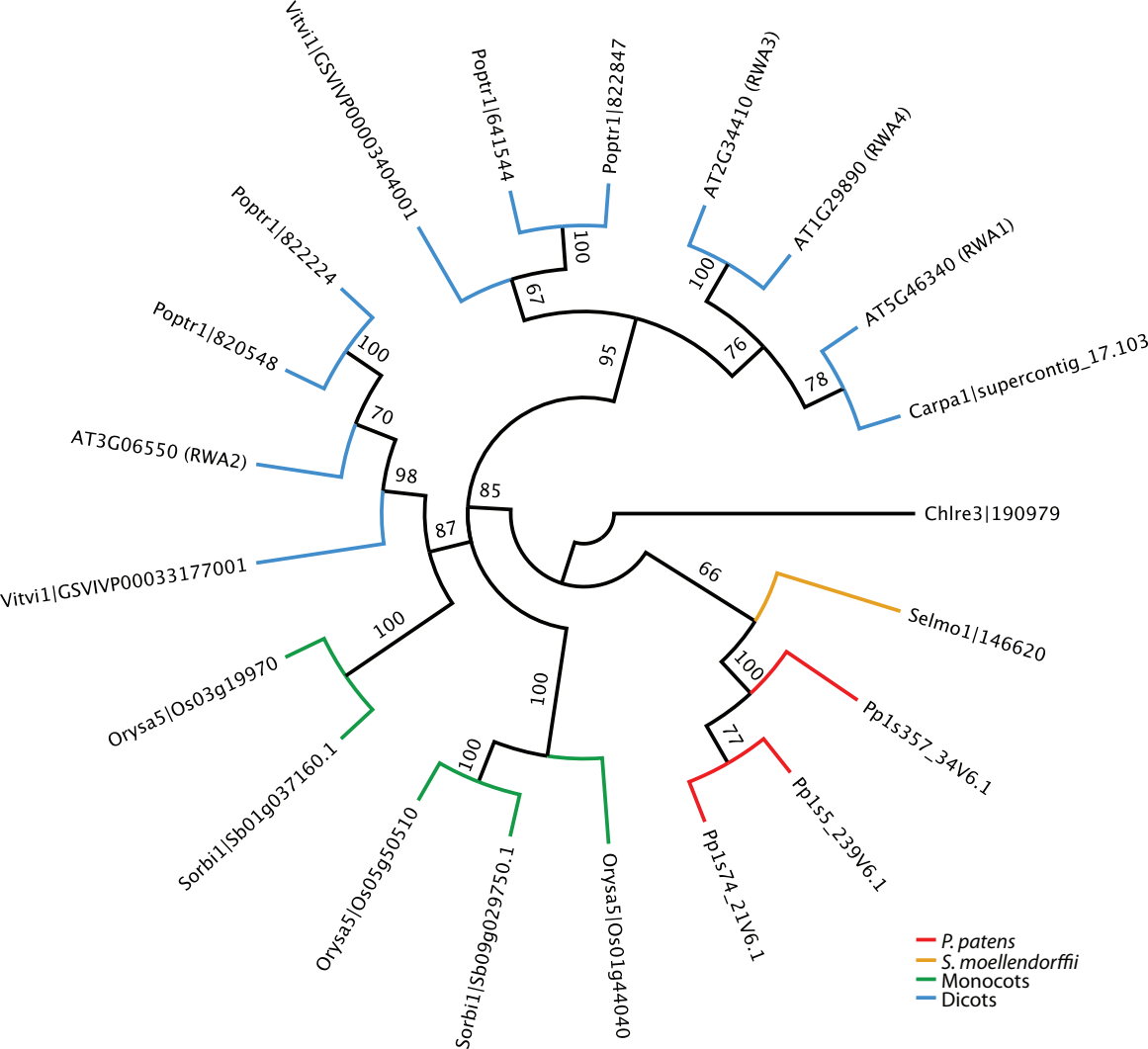

Supplement: Additional file 12: Figure S9 — Pectin acetyltransferase family tree. This family appears to be land plant-wide and is rooted by a gene from C. reinhardtii. The grouping of all the Physcomitrella genes into one monophyletic clade implies that there was only one family member in the common ancestor. [file 1471-2229-14-79-S12.pdf]

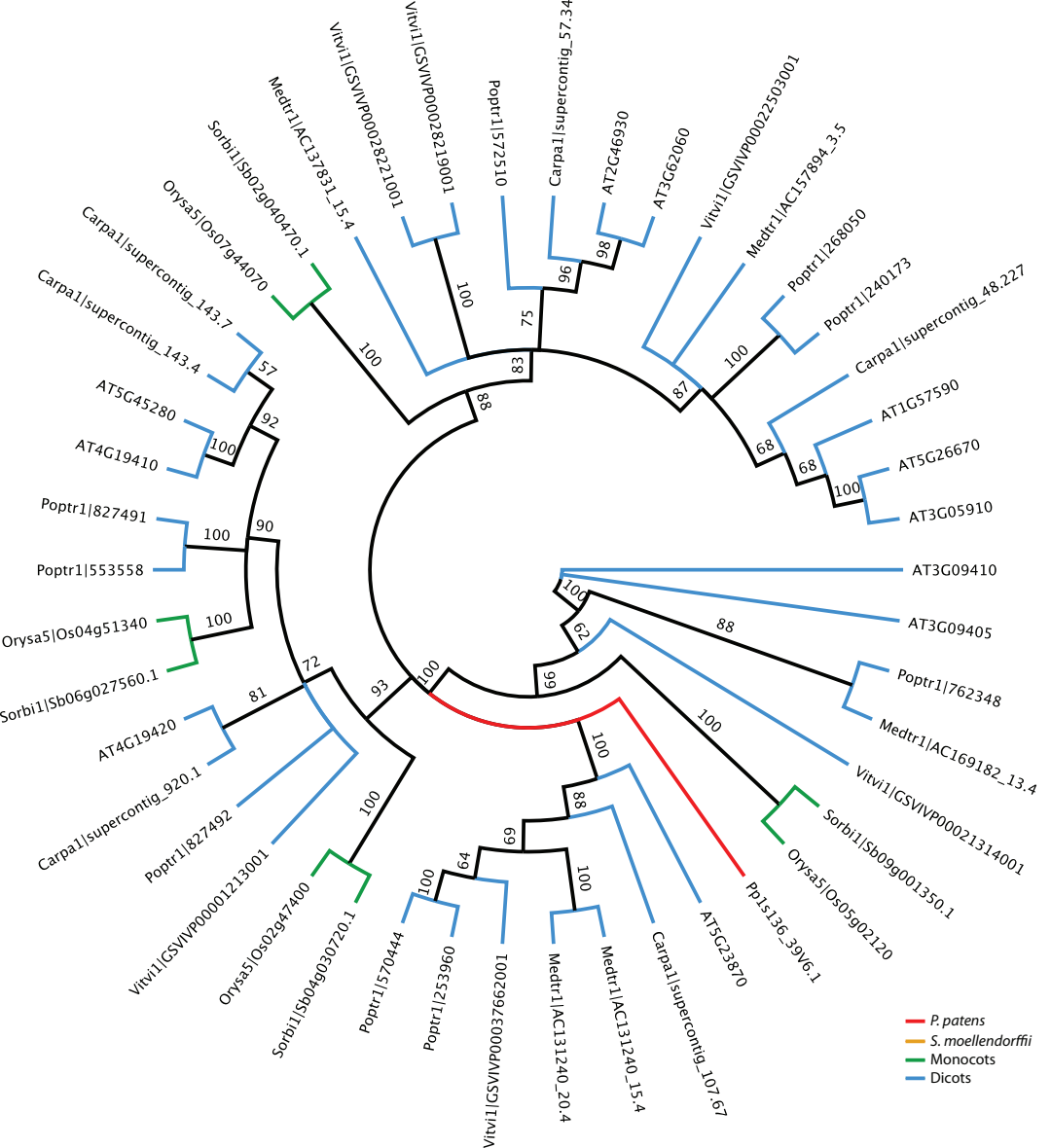

Supplement: Additional file 13: Figure S10 — Pectin acetylesterase family tree. This family contains only one Physcomitrella and no Selaginella members. [file 1471-2229-14-79-S13.pdf]

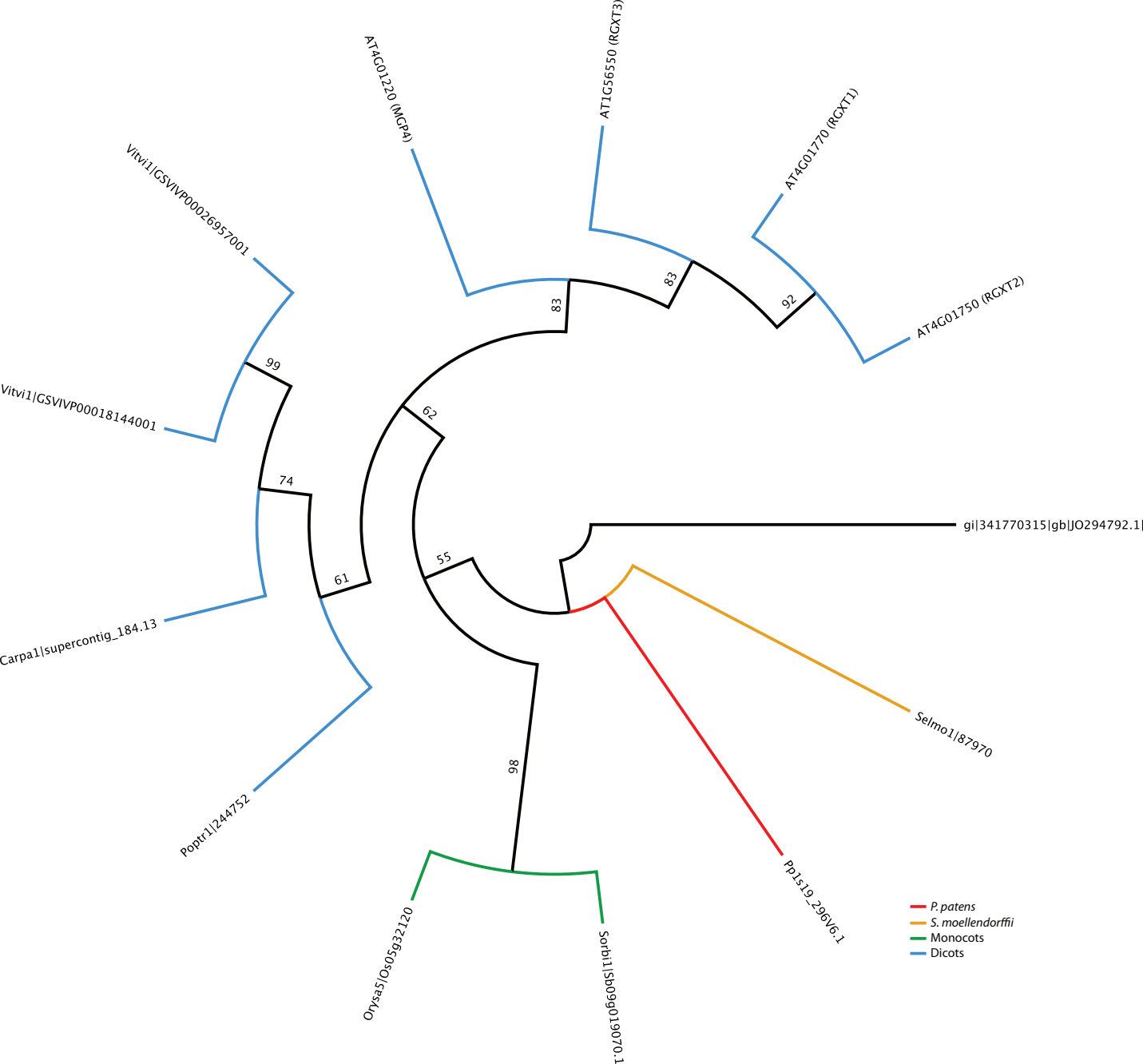

Supplement: Additional file 14: Figure S11 — Rhamnogalacturonan II xylosyltransferase family tree. This family appears to be land plant-wide, with one member in the common ancestor of land plants. The algal root gene is from Nitella hyalina. [file 1471-2229-14-79-S14.pdf]

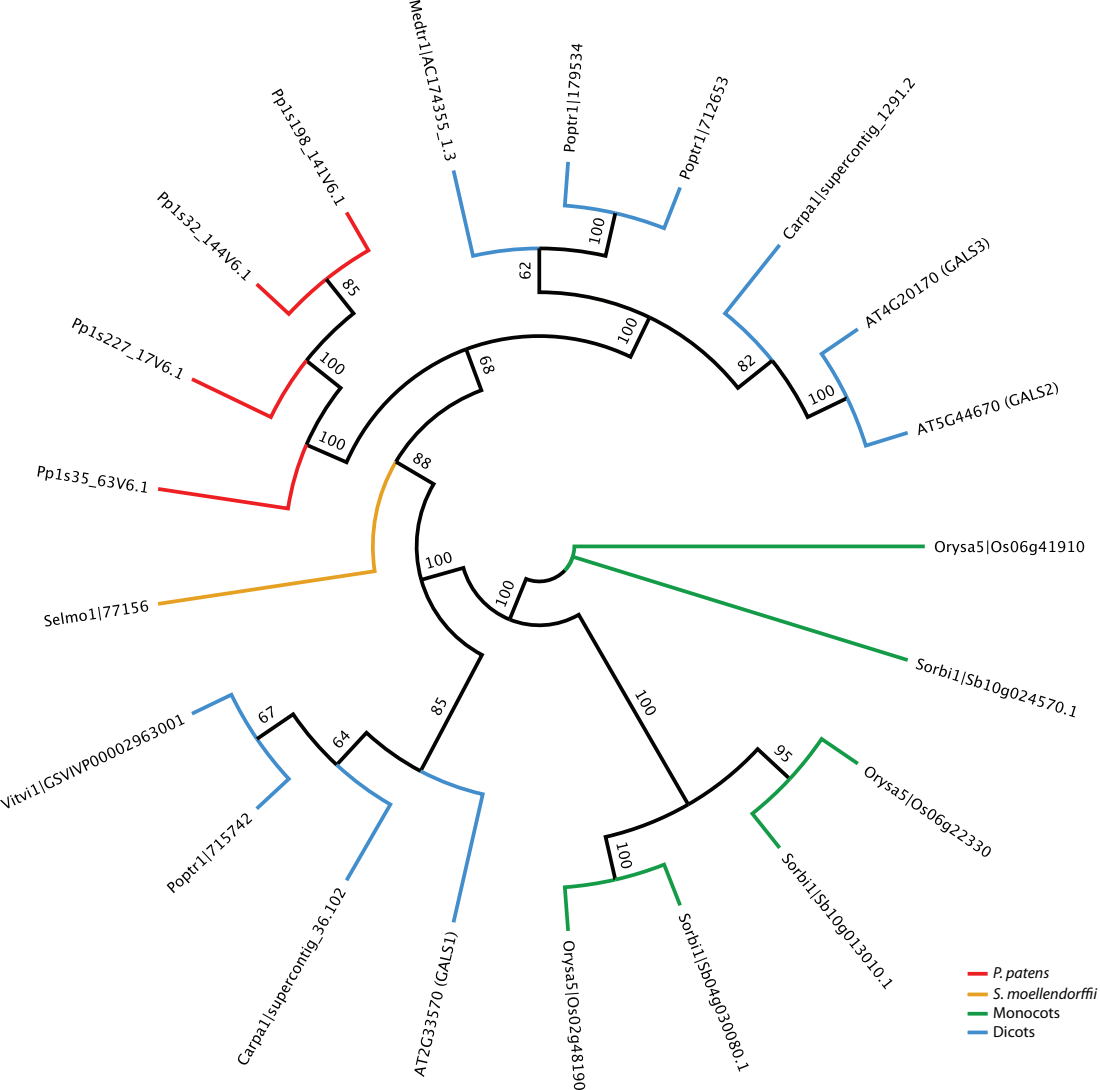

Supplement: Additional file 15: Figure S12 — β-1,4-Galactan β-1,4-Galactosyltransferase family tree. This tree has no algal root. The Physcomitrella genes are grouped together in a well-supported clade separate from other species. There is no evidence for more than one gene in the common ancestor. [file 1471-2229-14-79-S15.pdf]

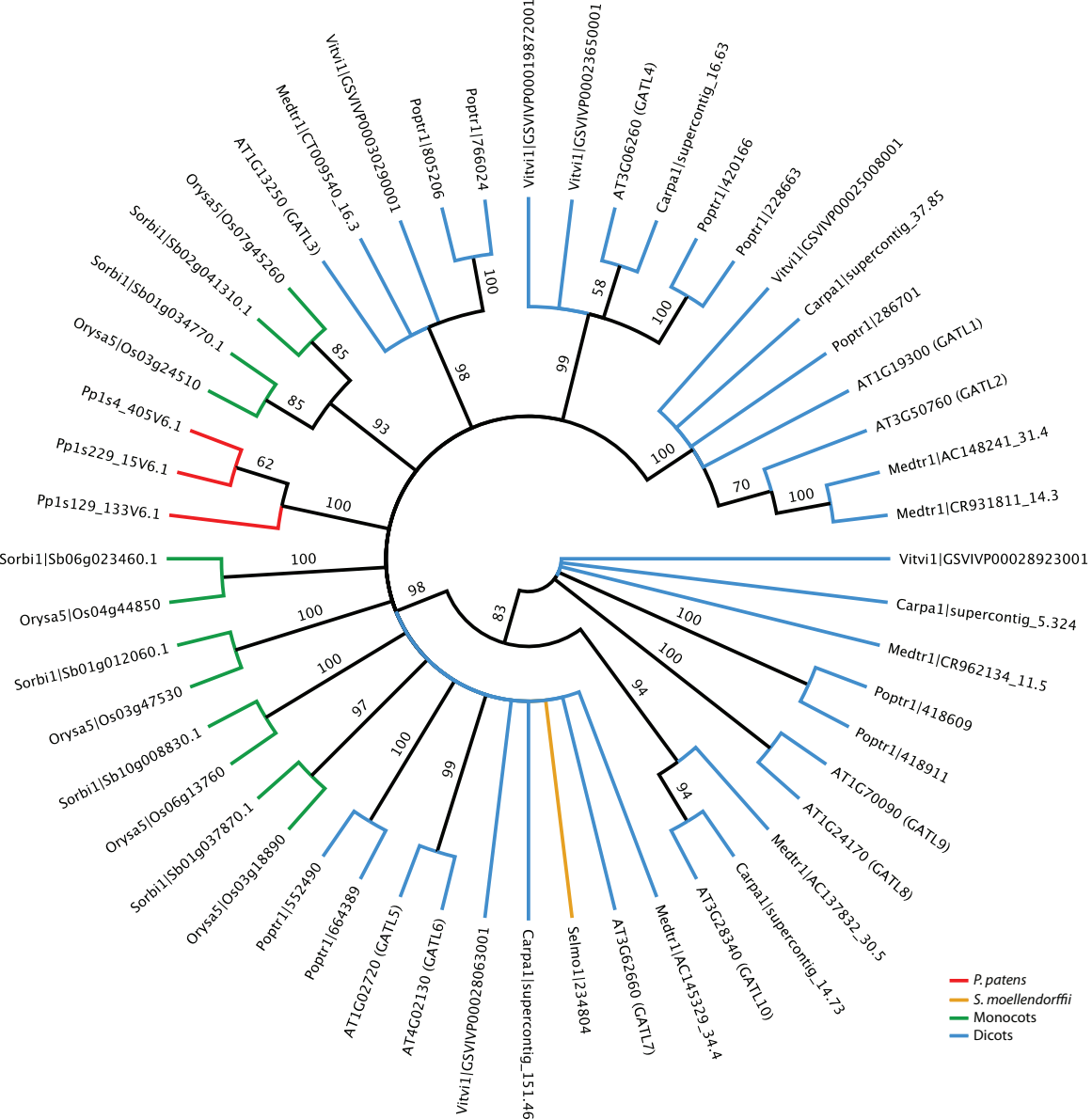

Supplement: Additional file 16: Figure S13 — GATL family tree. This tree is poorly resolved, with no root and large polytomies. The Physcomitrella genes group together in one well-supported clade. [file 1471-2229-14-79-S16.pdf]
